# Osteocrin attenuates inflammation, oxidative stress, apoptosis, and cardiac dysfunction in doxorubicin‐induced cardiotoxicity

**DOI:** 10.1002/ctm2.124

**Published:** 2020-07-03

**Authors:** Can Hu, Xin Zhang, Ning Zhang, Wen‐Ying Wei, Ling‐Li Li, Zhen‐Guo Ma, Qi‐Zhu Tang

**Affiliations:** ^1^ Department of Cardiology Renmin Hospital of Wuhan University Wuhan P. R. China; ^2^ Hubei Key Laboratory of Metabolic and Chronic Diseases Wuhan P. R. China

**Keywords:** apoptosis, doxorubicin, inflammation, osteocrin, oxidative stress

## Abstract

**Background:**

Inflammation, oxidative stress, and apoptosis contribute to the evolution of doxorubicin (DOX)‐induced cardiotoxicity. Osteocrin (OSTN) is a novel secretory peptide mainly derived from the bone and skeletal muscle, and plays critical roles in regulating bone growth and physical endurance. Inspiringly, OSTN was also reported to be abundant in the myocardium that functioned as a therapeutic agent against cardiac rupture and congestive heart failure in mice after myocardial infarction. Herein, we investigated the role and potential mechanism of OSTN in DOX‐induced cardiotoxicity.

**Methods:**

Cardiac‐restrict OSTN overexpression was performed by the intravenous injection of a cardiotropic AAV9 vector, and subsequently the mice received 15 mg/kg DOX injection (i.p., once) to induce acute cardiac injury. Besides, H9C2 cell lines were used to assess the possible role of OSTN in vitro by incubating with recombinant human OSTN or small interfering RNA against *Ostn* (si*Ostn*). To clarify the involvement of protein kinase G (PKG), KT5823 and si*Pkg* were used in vivo and in vitro. Mice were also administrated intraperitoneally with 5 mg/kg DOX weekly for consecutive 3 weeks at a cumulative dose of 15 mg/kg to mimic the cardiotoxic effects upon chronic DOX exposure.

**Results:**

OSTN treatment notably attenuated, whereas OSTN silence exacerbated inflammation, oxidative stress, and cardiomyocyte apoptosis in DOX‐treated H9C2 cells. Besides, cardiac‐restrict OSTN‐overexpressed mice showed an alleviated cardiac injury and malfunction upon DOX injection. Mechanistically, we found that OSTN activated PKG, while PKG inhibition abrogated the beneficial effect of OSTN in vivo and in vitro. As expected, OSTN overexpression also improved cardiac function and survival rate in mice after chronic DOX treatment.

**Conclusions:**

OSTN protects against DOX‐elicited inflammation, oxidative stress, apoptosis, and cardiac dysfunction via activating PKG, and cardiac gene therapy with OSTN provides a novel therapeutic strategy against DOX‐induced cardiotoxicity.

## Graphical abstract

1. OSTN alleviates DOX‐induced inflammation, oxidative stress and cardiomyocyte apoptosis in vivo and in vitro. 2. OSTN deficiency exacerbates DOX‐induced inflammation, oxidative stress and apoptosis in vitro. 3. OSTN overexpression attenuates DOX‐induced acute and chronic cardiotoxicity in mice. 4. The beneficial effects of OSTN against DOX‐induced cardiotoxicity are mediated by PKG activation.

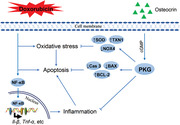

## 1 BACKGROUND

Doxorubicin (DOX) is one of the most widely prescribed antineoplastic drugs introduced over the past 50 years and remains the cornerstone for other targeted agents in standard tumor chemotherapy regimens. However, its therapeutic value and clinical use are extremely hampered due to the undesirable cardiotoxicity that eventually leads to congestive heart failure and cardiac death.^1^, ^2^, ^3^ Despite the efficiency in alleviating DOX‐elicited cardiac damage, long‐term application of dexrazoxane is related to a compromised chemotherapeutic activity, higher latent risk for new tumors, and early demise.^4^ Therefore, developing a safe and effective strategy to prevent DOX‐induced cardiotoxicity is highly desirable.

Inflammation, oxidative stress, and subsequent cardiomyocyte apoptosis are implicated in the evolution of DOX‐elicited cardiac dysfunction.^5^, ^6^, ^7^, ^8^, ^9^ Upon DOX injury, nuclear factor‐κB (NF‐κB) is activated within 1 h in the myocardium, where it triggers the synthesis of multiple inflammatory cytokines, such as tumor necrosis factor‐alpha (TNF‐α) and interleukin‐1 beta (IL‐1β).^10^, ^11^ These inflammatory cytokines then orchestrate a pro‐inflammatory microenvironment and recruit the infiltration of leukocytes into the myocardium to further exacerbate inflammatory injury. Accordingly, cardiac functional parameters were reported to negatively correlate with myocardial TNF‐α expression, and our previous study also proved that controlling cardiac inflammation was sufficient to attenuate cardiomyocyte death and cardiac dysfunction upon DOX injection.^6^, ^11^ In addition, DOX shows a high affinity to the iron and the formation of DOX‐iron complexes triggers unstrained reactive oxygen species (ROS) generation through a Fenton‐type reaction.^12^ DOX‐derived semiquinone free radicals also increase superoxide anions generation in the presence of molecular oxygen.^13^ ROS overproduction subsequently provokes oxidative damage to lipid/protein/nucleic acid and leads to cell apoptosis.^5^, ^9^ Of note, oxidative stress also directly promotes inflammatory cytokines expression and leukocyte chemotaxis, thereby amplifying the inflammatory processes.^14^, ^15^ Conversely, free radicals production can be enhanced by inflammation through Toll‐like receptor (TLR)‐dependent manners in DOX‐induced cardiotoxicity.^10^, ^11^ These studies provide a basis for targeting inflammation and oxidative stress as an effective therapeutic strategy to ameliorate DOX‐induced cardiotoxicity.

Osteocrin (OSTN), also known as musclin, is initially proposed as a novel secretory peptide released mainly from the bone and skeletal muscle, and plays critical roles in regulating bone growth and physical endurance.^16^, ^17^ Data from Lanctôt's laboratory indicated that OSTN treatment significantly blunted the terminal differentiation of osteoblasts and promoted the proliferation of chondrocytes in growth plate, whereas Subbotina et al determined that OSTN functioned as an exercise‐responsive myokine to enhance the mitochondrial biogenesis and exercise tolerance.^18^, ^19^, ^20^ Except for the pathophysiological role in the bone and skeletal muscle, recent studies clarified that OSTN might also be implicated in heart diseases. Chiba et al previously revealed that OSTN was abundantly expressed in the myocardium and could be secreted from the cardiomyocytes.^21^ Furthermore, a recent study found that OSTN treatment notably suppressed heart failure and cardiac rupture in mice after myocardial infarction.^22^ Protein kinase G (PKG) serves as a cyclic guanosine monophosphate (cGMP)‐dependent serine/threonine protein kinase that is required for controlling inflammation, oxidative stress, and cardiomyocyte apoptosis in various cardiac diseases.^23^, ^24^ As expected, PKG activation notably alleviates DOX‐triggered cardiac injury and malfunction, accompanied with an increased chemotherapeutic efficacy.^25^, ^26^, ^27^ Previous studies indicated that OSTN treatment was sufficient to elevate cGMP levels and PKG activity, however, its pathophysiological role in DOX‐induced cardiotoxicity has remained elusive.^19^, ^20^, ^21^

## 2 MATERIALS AND METHODS

### 2.1 Reagents

The following primary antibodies were obtained from Abcam (Cambridge, UK): anti‐NADPH oxidase 2 (NOX2, #ab129068), anti‐NOX4 (#ab109225), anti‐superoxide dismutase 2 (SOD2, #ab13533), anti‐B cell lymphoma 2 (BCL‐2, #ab196495), anti‐cell differentiation factor 45 (CD45, #ab10558), anti‐CD206 (#ab8918), and anti‐CD68 (#ab53444), whereas anti‐glyceraldehyde 3‐phosphate dehydrogenase (GAPDH, #2118), anti‐BCL‐2‐associated X protein (BAX, #2772), anti‐phospho‐NF‐κB P65 (P‐P65, #3033), anti‐total‐NF‐κB P65 (T‐P65, #8242), anti‐P‐vasodilator‐stimulated phosphoprotein (P‐VASP, #3114), and anti‐T‐VASP (#3132) were obtained from CST (Danvers, USA). Anti‐proliferating cell nuclear antigen (PCNA, #sc‐7907) and anti‐CD80 (#sc‐376012) were purchased from Santa Cruz Biotechnology (Dallas, USA). DOX and the ELISA kits for IL‐1β and TNF‐α were obtained from Sigma–Aldrich (St. Louis, USA). Carrier‐free recombinant human OSTN protein (rhOSTN) was obtained from R&D Systems (Minneapolis, USA). Dihydroethidium (DHE) and 2′,7′‐dichlorodihydrofluorescein diacetate (DCFH‐DA) were purchased from Nanjing KeyGen (Jiangsu, China) or Beyotime Biotechnology (Shanghai, China), respectively. Cyclic GMP XP^®^ assay kit was obtained from CST, while cGMP‐dependent protein kinase assay kit was purchased from CycLex^®^ (Nagano, Japan). KT5823, a potent and selective inhibitor for PKG and 3‐nitrotyrosine (3‐NT) ELISA assay kit were obtained from Abcam. Assay kits for detecting malondialdehyde (MDA) content, total SOD activity, and NOX activity were obtained from Nanjing Jiancheng Bioengineering Institute (Nanjing, China). EnzChek™ caspase3 assay kit and cell counting kit‐8 (CCK‐8) were purchased from ThermoFisher Scientific (Waltham, USA) or Dōjindo Laboratories (Kumamoto, Japan), respectively. Terminal deoxynucleotidyl transferase‐mediated dUTP‐biotin nick end labeling (TUNEL) assay kit was purchased from Millipore (Billerica, USA). Small interfering RNA against *Ostn* (si*Ostn*) or PKG (si*Pkg*) and their scramble RNA (si*RNA*) were generated by RiboBio (Guangdong, China), and the adeno‐associated virus 9 (AAV9) vectors containing a cardiac‐specific cTnT promoter were synthesized by DesignGene Biotechnology (Shanghai, China). The full length of OSTN was cloned into the AAV9 shuttle plasmid under the control of cTnT promoter to generate AAV9‐OSTN, whereas the blank AAV9 vector without any tags were regarded as the negative control (AAV9‐NC).

### 2.2 Experimental animals and treatments

Adult male C57/B6 mice (8‐10 weeks old) were purchased from the ILAS, CAMS, and PUMC (Beijing, China) and then bred in a SPF barrier system under a regular 12 h light/dark cycle, with free access to a standard laboratory chow diet. After acclimation for 1 week, mice were subjected to a single tail vein injection (1 × 10^11^ viral genome/mouse) of AAV9‐OSTN or AAV9‐NC according to our previous studies.^5^, ^28^ Four weeks later, mice received a single bolus injection of DOX (15 mg/kg, intraperitoneally [i.p.]) to induce acute cardiac injury, while the normal saline (NS) was injected as a negative control.^5^, ^6^ Mice were maintained for 8 days and received functional measurements at the study termination, which were then euthanized immediately with the heart excised for further study. To verify the role of PKG, mice were i.p. injected with KT5823 (1 mg/kg/day) for consecutive 3 days prior to DOX treatment via referring to a previous study.^29^ DOX‐induced cardiac injury appears acutely within several days of exposure in ∼11% of patients but also after chronic treatment in about 1.7% of the cases.^30^, ^31^ Therefore, mice were also administrated i.p. with 5 mg/kg DOX weekly for consecutive 3 weeks at a cumulative dose of 15 mg/kg to mimic the cardiotoxic effects upon chronic DOX exposure.^5^, ^6^ Four weeks from the last DOX injection, functional parameters were recorded and heart samples were excised for the next study. Overall, Our DOX dosing protocol at 15 mg/kg in mice is a dose that allometrically scales to roughly 45 mg/m^2^ in humans, within the limits of the current therapeutic regimens (30‐90 mg/m^2^) in clinic.^32^, ^33^ All animal protocols were approved by the Animal Care and Use Committee of Renmin Hospital of Wuhan University. Randomization procedures and blinded methods ran through the whole experiments.

### 2.3 Cardiac function analysis

Echocardiographic images were captured from 1.5% isoflurane‐anesthetized mice using a previously described MyLab 30CV machine (Esaote SpA) with a 10‐MHz probe.^34^, ^35^, ^36^ The parameters were then calculated and averaged from at least five consecutive cardiac cycles based on the M‐mode images. Hemodynamic monitoring was performed by the 1.4F Millar catheter‐tip micromanometer catheter (SPR‐839; Millar Instruments, Houston, TX) linked to a PowerLab system as previously described.^35^, ^37^ Hemodynamic parameters were analyzed using the PVAN software.

### 2.4 Quantitative real‐time PCR and western blot

Quantitative real‐time PCR and western blot were conducted as our previously described.^37^, ^38^, ^39^ After being extracted by the TRIzol reagent, total RNA was converted to cDNA using oligo dT primers that served as a template to assess the relative gene expression on the Roche LightCycler^®^ 480 detection system using SYBR green. Proteins were extracted from ventricules or cells and the concentrations were determined by a BCA kit. Fifty micrograms of the proteins were exposed to SDS‐PAGE (30% acrylamide) separation and then transferred onto PVDF membranes. After the incubation with indicating primary antibodies (4°C, overnight) and secondary antibodies (room temperature, 1 h), respectively, the membranes were then visualized by a ChemiDoc™ XRS+ chemiluminescence system and analyzed using an Image Lab Software (Bio‐Rad Laboratories, Inc.).

### 2.5 Mitochondrial content and adenosine triphosphate measurement

Mitochondrial genomic DNA (mtDNA) and protein (mtProtein) levels were used to evaluate mitochondrial content according to previous studies.^20^, ^40^ DNA was purified from heart samples using a commercial DNeasy kit and mtDNA copy number was calculated from the normalized Ct values of MT‐CO2 gene (encoded by mtDNA), whereas mtDNA integrity was determined by the ratio of long to short fragments. Mitochondria were isolated from heart samples using a mitochondria isolation kit and then mtProtein level was normalized to the wet tissue weight. Cardiac ATP level was measured by a commercial ATP assay kit as our previously described.^41^ In brief, heart samples were lysed and centrifuged (4°C, 5 min) with the supernatants incubated with the ATP detection working solution. Then the luminescence values were recorded and normalized to total proteins.

### 2.6 Histological analysis

Immunofluorescence and TUNEL staining were performed according to previous studies.^8^, ^34^, ^38^ Briefly, cardiac slices were deparaffinized by dimethylbenzene and rehydrated in graded ethanol solution, which were then probed by (4°C, overnight) the primary antibodies after citrate‐mediated high‐temperature antigen retrieval process. Next, slices were stained with (37°C, 1 h) Alexa Fluor 488 (Green) or Alexa Fluor 568 (Red)‐conjugated antibodies, followed by DAPI incubation in the dark. For immunofluorescence staining in cells, coverslips were fixed and permeabilized using 4% paraformaldehyde (15 min) or 0.2% Triton X‐100 (5 min) at room temperature, respectively. The cells were then stained with (4°C, overnight) anti‐T‐P65 (1:100 dilution) and Alexa Fluor 568‐labeled antibody (1:200 dilution; 37°C, 1 h), followed by DAPI incubation in the dark. TUNEL staining was performed according to the manufacturer's instructions as our previously described. Images were acquired via a fluorescence microscope (DX51, Olympus).

### 2.7 Biochemical analysis

Serum levels of lactate dehydrogenase (LDH), cardiac isoform of Tropnin T (cTnT), and creatine kinase isoenzymes (CK‐MB) were detected by an automatic biochemical analyzer (ADVIA^®^ 2400; Siemens) as previously described.^5^, ^7^ IL‐1β, TNF‐α, cGMP, MDA, and 3‐NT levels in the myocardium or cell medium were tested by the commercially available ELISA kits.^22^, ^24^ Cell viability was assessed by the CCK‐8 method in vitro via referring to our previous studies.^5^, ^6^ Fasting blood glucose (FBG) and fasting insulin levels (FIns) were detected by an automatic glucometer and the commercial insulin ELISA kit, respectively. The homeostasis model of assessment for insulin resistance (HOMA‐IR) index was calculated to further evaluate insulin resistant status. Circulating OSTN level was detected using the sandwich chemiluminescence enzyme immunoassay (CLEIA) as previously described.^17^, ^22^ In brief, mouse blood samples were centrifuged at 4°C for 20 min and applied to precoated plates at 4°C overnight, followed by the incubation with an anti‐mouse/human OSTN rat antibody at room temperature for 1 h. Next, alkaline phosphatase‐conjugated donkey anti‐rat antibody was added to incubate for additional 1 h, which was then detected at 535 nm using the CDP‐Star™ substrate with Emerald‐II™ enhancer.

### 2.8 Cell experiments

H9C2 cell lines were cultured in DMEM medium containing 10% FBS to achieve 50‐60% confluency and then were treated with rhOSTN (5µg/mL) or vehicle in the presence or absence of DOX (1µmol/L) for 24 h after synchronization.^5^, ^7^ In silence studies, cells were pre‐infected with si*Ostn* (50 nmol/L), si*Pkg* (50 nmol/L), or si*RNA* (50 nmol/L) for 4 h using Lipo6000, and then maintained in normal medium for additional 24 h before further stimulation according to our previous studies.^5^, ^7^

### 2.9 DHE and DCFH‐DA staining

DHE and DCFH‐DA staining were performed to evaluate ROS level in heart samples or cells respectively according to our previous studies.^5^, ^7^, ^38^ In brief, fresh frozen cardiac slices and H9C2 cells were stained (37°C, 30 min) with DHE (5 µmol/L) or DCFH‐DA (5 µmol/L) in the dark and then were visualized under the Olympus IX53 fluorenscence microscope (Tokyo, Japan).

### 2.10 Enzymatic activities detection

Total SOD and NOX activities were determined using the commercially available kits as our previously described.^5^, ^38^ Caspase3 activity in the myocardium or cultured cells was measured via detecting the fluorogenic change of Z‐DEVD‐AMC as previously described.^42^ PKG activity in hearts or cells was assayed by colorimetric method via referring to a previous study.^24^

### 2.11 Statistical analysis

Values are expressed as the mean ± standard deviation (SD) and analyzed by SPSS software (Version 22.0). Unpaired Student's *t*‐test was used to compare differences between two groups, whereas comparisons for more than two groups were performed by one‐way ANOVA analysis with Tukey post hoc test. Statistical significance was set at *P *< .05.

## 3 RESULTS

### 3.1 OSTN alleviates DOX‐induced inflammation in vitro

**FIGURE 1:**
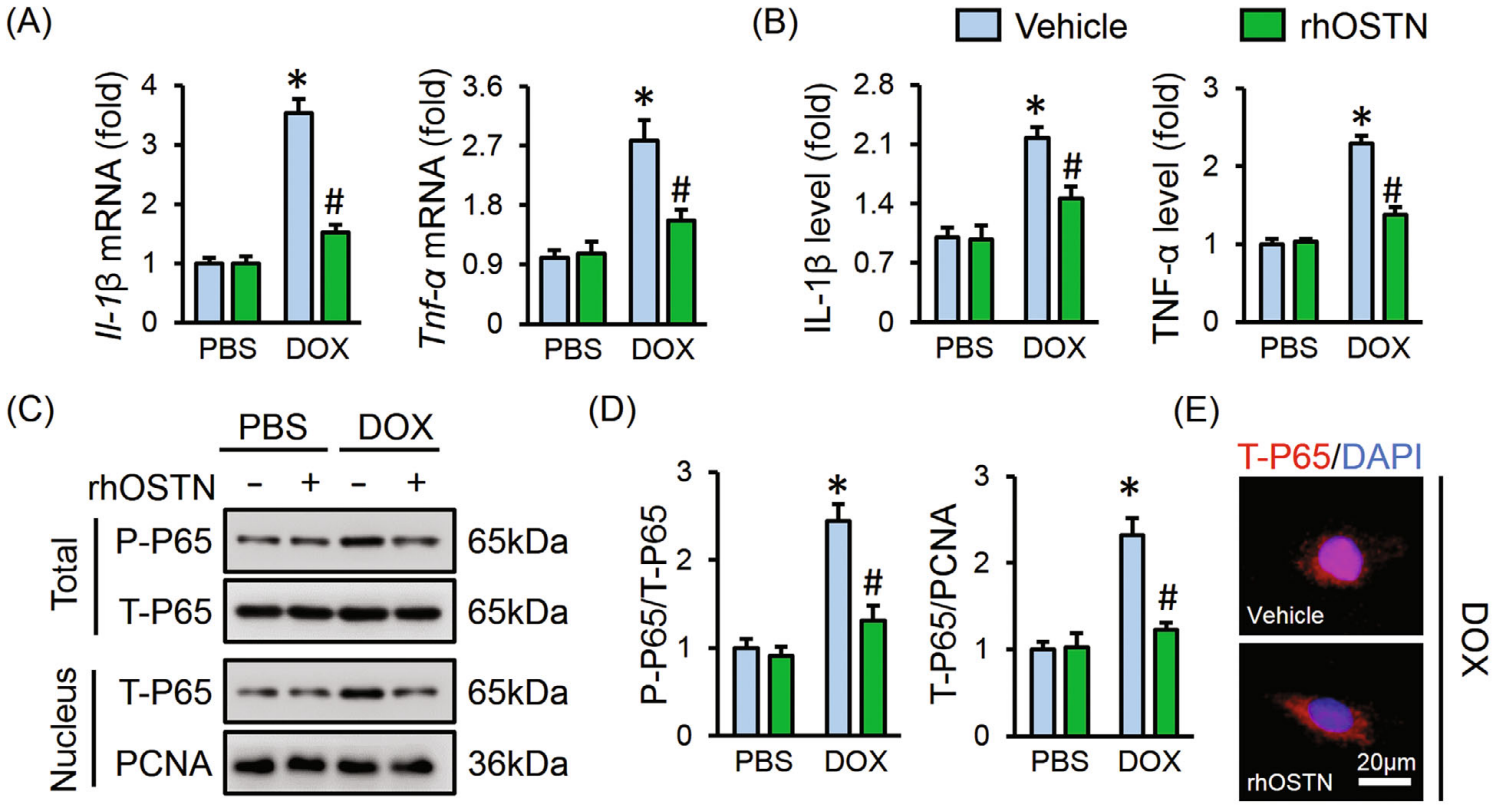
OSTN alleviates DOX‐induced inflammation in vitro. H9C2 cells were treated with rhOSTN (5 µg/mL) or an equal volume of vehicle as the control in the presence or absence of DOX (1 µmol/L) for 24 h. A, The mRNA levels of inflammatory markers, *Il‐1β* and *Tnf‐α* (n = 6). B, The releases of IL‐1β, TNF‐α from H9C2 cells to the medium (n = 6). C and D, Representative western blot images and the quantitative results (n = 6). E, Immunofluorescence staining of T‐P65 in H9C2 cells (n = 6). Values represent the mean ± SD. *P < .05 versus PBS + Vehicle; ^#^ *P *< .05 versus DOX + Vehicle

DOX injection is reported to trigger the production of a certain pro‐inflammatory cytokines in the myocardium that contribute to DOX‐induced cardiotoxicity.^6^, ^11^ As shown in Figure 1A,B, DOX incubation remarkably triggered the synthesis of IL‐1β and TNF‐α in H9C2 cells, and promoted their releases to the medium, which were markedly inhibited by rhOSTN treatment. We then detected the phosphorylation and nuclear translocation of P65 that acts as a key transcriptional regulator in inflammatory response. Western blot showed that DOX treatment promoted the phosphorylation and nuclear translocation of P65, whereas rhOSTN incubation significantly alleviated P65 activation upon DOX insult (Figure 1C,D). Immunofluorescence staining further revealed that OSTN prevented DOX‐induced P65 nuclear translocation (Figure 1E). Therefore, we summarize that OSTN alleviates DOX‐induced inflammatory response in vitro.

### 3.2 OSTN attenuates DOX‐induced oxidative stress in vitro

**FIGURE 2:**
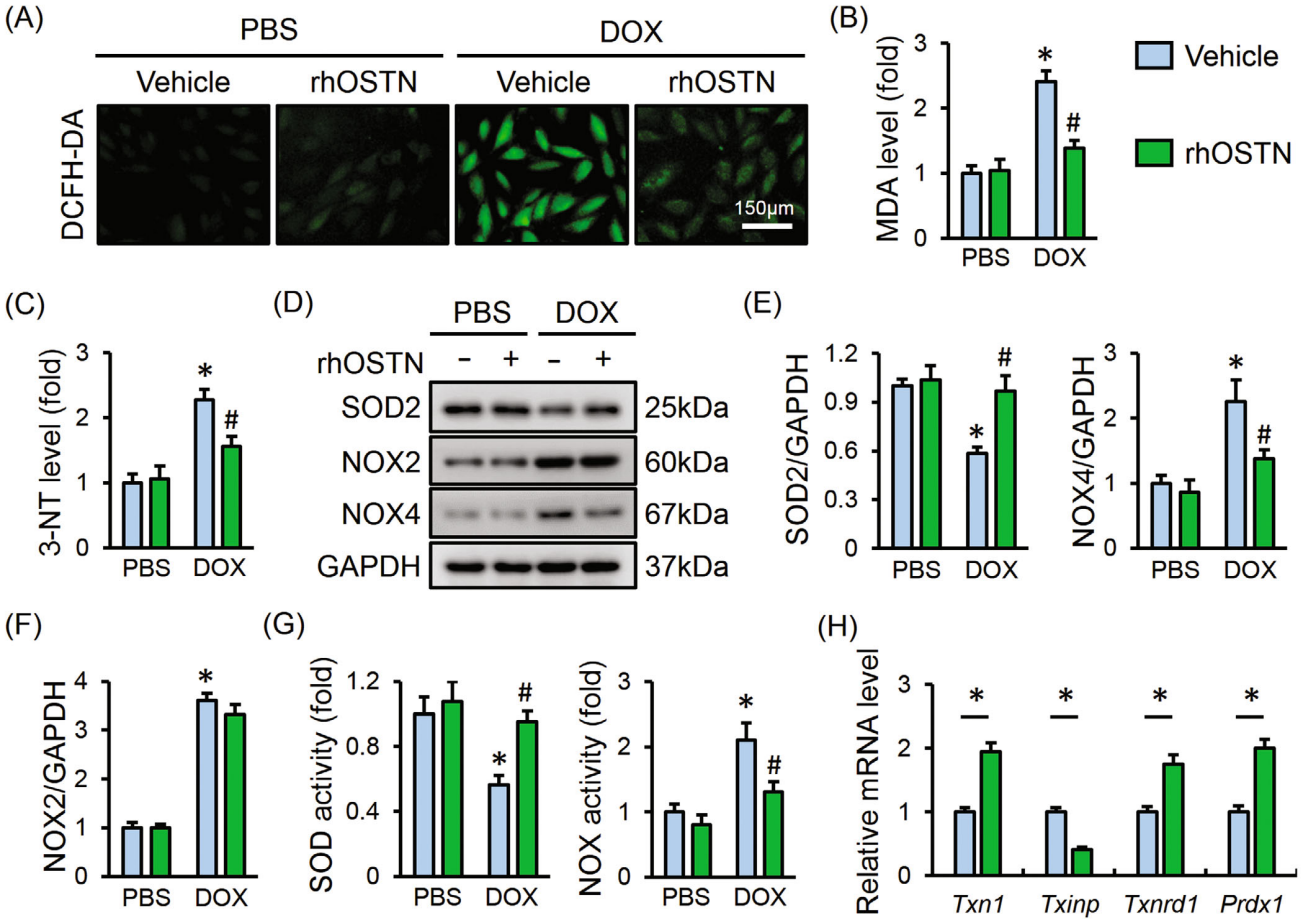
OSTN attenuates DOX‐induced oxidative stress in vitro. A, Representative images of DCFH‐DA staining in H9C2 cells treated with rhOSTN in the presence or absence of DOX (n = 6). B and C, The level of MDA and 3‐NT in H9C2 cells (n = 8). D‐F, Representative western blot images and the corresponding statistical results (n = 6). G, Total SOD activity and NOX activity in H9C2 cells (n = 8). H, The mRNA level of *Txn1*, *Txinp*, *Txnrd1*, and *Prdx1* in DOX‐treated H9C2 cells with or without rhOSTN protection (n = 6). Values represent the mean ± SD. ^*^ *P *< .05 versus PBS + Vehicle; ^#^ *P *< .05 versus DOX + Vehicle. In Figure 2H, ^*^ *P *< .05 versus the matched group

It is well accepted that oxidative damage is closely implicated in DOX‐elicited cardiac injury, therefore, H9C2 cells were stimulated by DOX with or without rhOSTN incubation. DCFH‐DA staining indicated that DOX‐triggered ROS generation was markedly attenuated by OSTN (Figure 2A). MDA and 3‐NT are identified as the biomarker for lipid or protein peroxidation, respectively. In line with the increased ROS level, MDA and 3‐NT generation were augmented in DOX‐treated H9C2 cells, which were notably suppressed by rhOSTN incubation (Figure 2B,C). As depicted in Figure 2D,F, rhOSTN treatment markedly increased SOD2 while decreased NOX4 protein levels in DOX‐stimulated H9C2 cells, without affecting NOX2 expression. Accordingly, DOX‐elicited downregulation of total SOD activity and upregulation of NOX activity were both prevented in cells with rhOSTN incubation (Figure 2G). Thioredoxin system is composed of the anti‐oxidant thioredoxin‐1 (TXN1), thioredoxin reductase (TXNRD1), thioredoxin peroxidase‐1 (PRDX1), and the pro‐oxidant thioredoxin‐interacting protein (TXINP), which functions as a crucial defense mechanism against oxidative damage in cardiovascular diseases.^43^, ^44^ Moreover, previous studies showed that thioredoxin‐mediated redox homeostasis was disturbed by DOX treatment, and TXN1 overexpression significantly attenuated DOX‐induced cardiotoxicity.^45^, ^46^, ^47^ Consistent with these data, we observed that the anti‐oxidant gene, *Txn1*, *Txnrd1*, and *Prdx1* were upregulated, whereas the pro‐oxidant gene *Txinp* was downregulated in cells treated with rhOSTN after DOX stimulation (Figure 2H). Collectively, these results imply that OSTN attenuates DOX‐induced oxidative stress in vitro.

### 3.3 OSTN protects against DOX‐induced cardiomyocyte apoptosis in vitro

**FIGURE 3:**
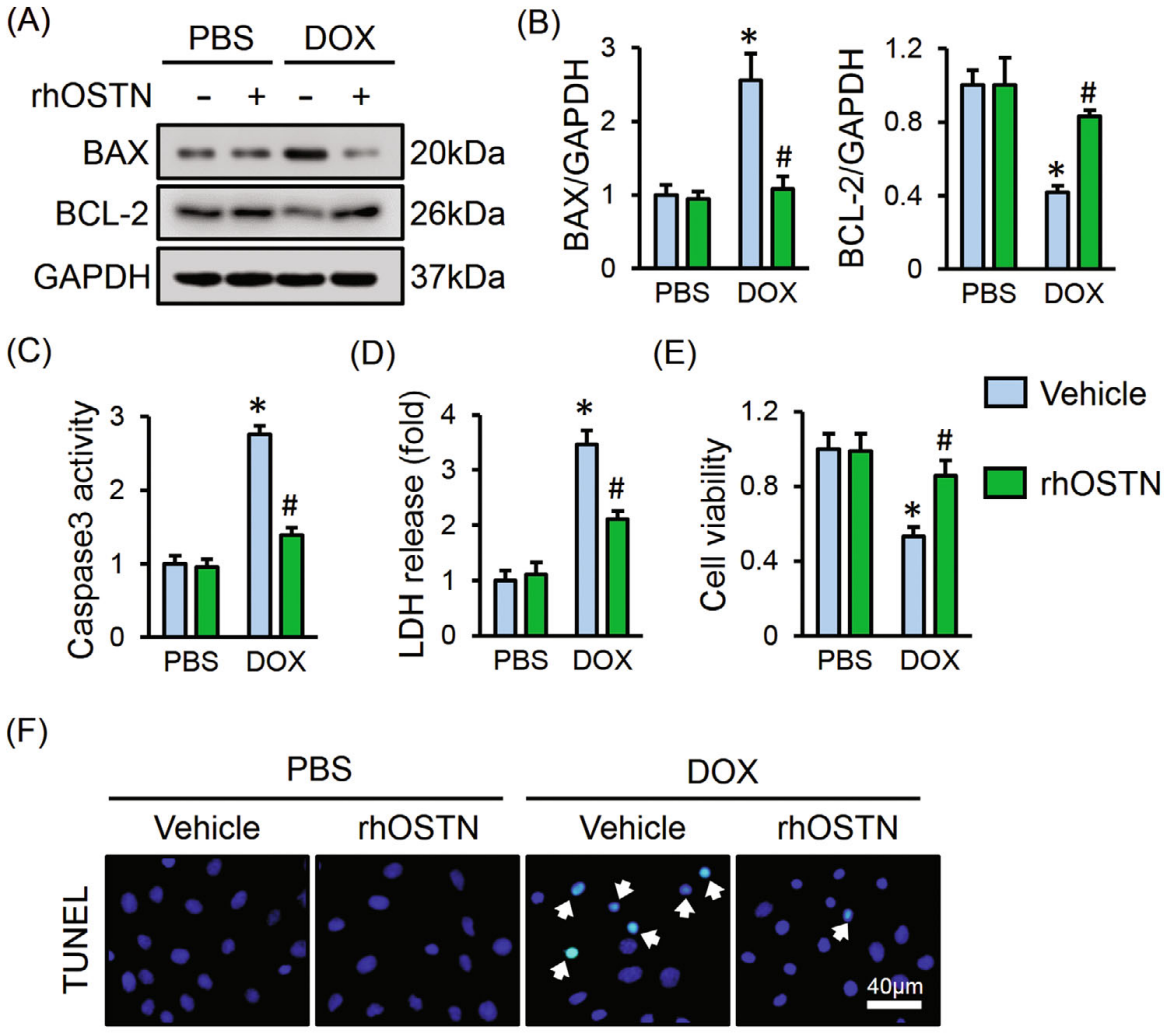
OSTN protects against DOX‐induced cardiomyocyte apoptosis in vitro. A and B, Representative western blot images and the corresponding statistical results (n = 6). C, Caspase3 activity in H9C2 cells (n = 6). D, LDH release from H9C2 cells (n = 6). E, Cell viability assessed by CCK‐8 kits (n = 8). F, Representative images of TUNEL staining in H9C2 cells (n = 6). Values represent the mean ± SD. ^*^ *P *< .05 versus PBS + Vehicle; ^#^ *P *< .05 versus DOX + Vehicle

Cardiomyocyte apoptosis is a hallmark in DOX‐induced cardiotoxicity, which can also be aggravated by the uncontrolled inflammation and oxidative stress. Consistently, we observed that DOX treatment evidently increased BAX expression and decreased BCL‐2 expression, which were blunted in the presence of rhOSTN (Figure 3A,B). Further detection showed that rhOSTN treatment also suppressed DOX‐elicited increase of caspase3 activity (Figure 3C). In accordance with the molecular alterations, DOX‐elicited cell injury and death were also improved by rhOSTN, as assessed by the decreased LDH release and increased cell viability (Figure 3D,E). The anti‐apoptotic capacity of OSTN was also directly determined by the TUNEL staining, showing a decreased TUNEL‐positive nuclei in rhOSTN‐treated cells (Figure 3F). All the data implicate that OSTN prevents DOX‐induced cardiomyocyte apoptosis in vitro.

### 3.4 Ostn deficiency exacerbates DOX‐induced inflammation, oxidative stress, and apoptosis in vitro

**FIGURE 4:**
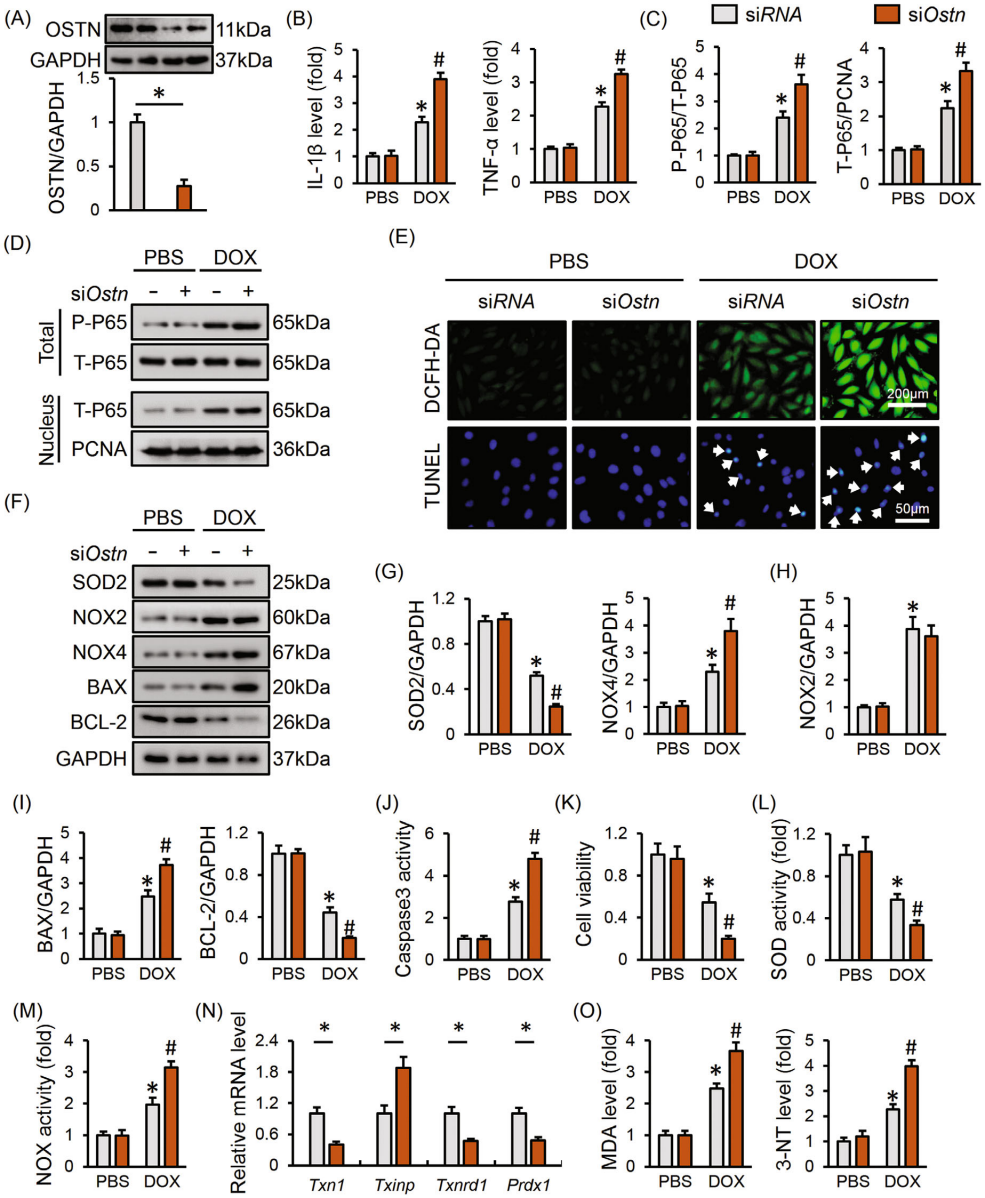
*Ostn* deficiency exacerbates DOX‐induced inflammation, oxidative stress and apoptosis in vitro. H9C2 cells were pre‐infected with si*Ostn* (50 nmol/L) or si*RNA* (50 nmol/L) for 4 h and then maintained in normal medium for 24 h before DOX (1 µmol/L) incubation for additional 24 h. A, The efficiency of si*Ostn* in H9C2 cells (n = 6). B, The IL‐1β and TNF‐α levels in the medium released from H9C2 cells (n = 6). C and D, Representative western blot images and the corresponding statistical results (n = 6). E, Representative images of DCFH‐DA and TUNEL stating (n = 6). F‐I, Representative western blot images and the corresponding statistical results (n = 6). J, Caspase3 activity in H9C2 cells (n = 8). K, Cell viability assessed by CCK‐8 kits (n = 8). L and M, SOD and NOX activity in H9C2 cells (n = 8). N, The mRNA level of *Txn1*, *Txinp*, *Txnrd1*, and *Prdx1* in H9C2 cells (n = 6). O, The level of MDA, 3‐NT in H9C2 cells (n = 8). Values represent the mean ± SD. ^*^ *P *< .05 versus PBS + si*RNA*; ^#^ *P *< .05 versus DOX + si*RNA*. In Figure 4A,N, ^*^ *P *< .05 versus the matched group

We then investigated whether *Ostn* deficiency affected inflammation, oxidative stress, and apoptosis upon DOX treatment in vitro. Cells with si*Ostn* infection had a significant decrease of OSTN protein level (Figure 4A). As depicted in Figure 4B, DOX‐elicited IL‐1β and TNF‐α releases from H9C2 cells were augmented with *Ostn* silence. Meanwhile, P65 phosphorylation and nuclear translocation were enhanced in *Ostn*‐deficient cells (Figure 4C,D). In addition to inflammation, DOX‐triggered ROS generation and cell apoptosis were also aggravated in cells with OSTN knockdown, as evidenced by the DCFH‐DA and TUNEL staining (Figure 4E). Consistently, *Ostn* silence further downregulated SOD2, BCL‐2 level and upregulated NOX4, BAX level in response to DOX injury, with no alteration on NOX2 protein level (Figure 4F,I). DOX‐triggered increase of caspase3 activity and decrease of cell viability were also augmented in *Ostn*‐deficient cells (Figure 4J,K). Besides, OSTN knockdown remarkably suppressed SOD activity and increased NOX activity in DOX‐treated H9C2 cells (Figure 4L,M). The anti‐oxidant thioredoxin system was further disturbed in *Ostn*‐deleted cells after DOX incubation, as evidenced by the decreased *Txn1*, *Txnrd1*, *Prdx1*, and increased *Txinp* mRNA levels (Figure 4N). Accordingly, we observed that MDA and 3‐NT production were markedly increased in si*RNA*‐treated cells but to a more prominent extent in si*Ostn*‐infected cells after DOX insult (Figure 4O). However, OSTN expression pattern appeared to not affect inflammation, oxidative stress, and cardiomyocyte apoptosis under basal conditions (Figure 4A‐O). These data provide solid evidence that OSTN deletion promotes inflammation, oxidative stress, and apoptosis upon DOX insult.

### 3.5 OSTN suppresses DOX‐induced inflammatory response in vivo

**FIGURE 5:**
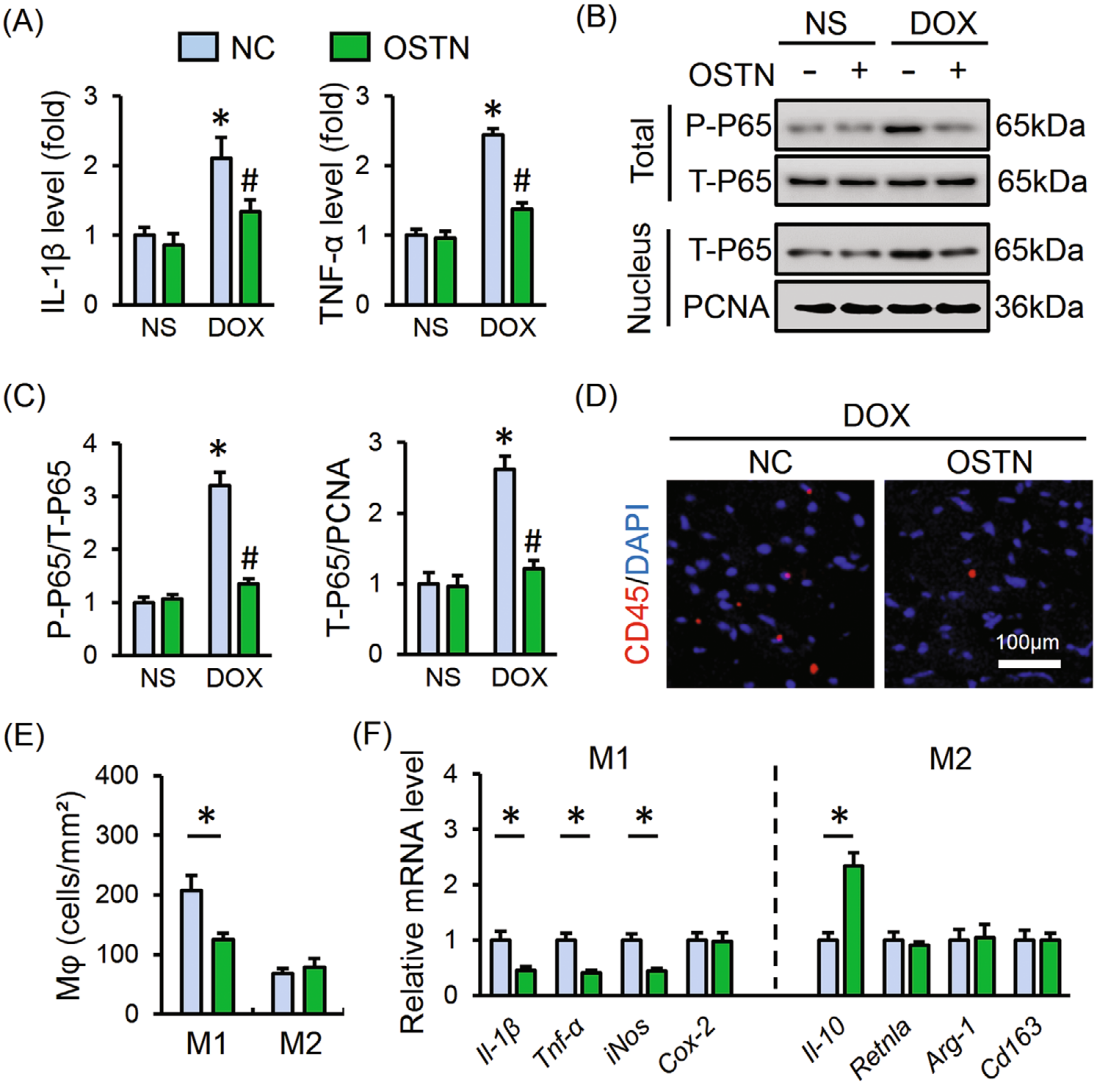
OSTN suppresses DOX‐induced inflammatory response in vivo. Mice were exposed to a single intravenous injection of AAV9‐OSTN (OSTN) or AAV9‐NC (NC) at a dosage of 1 × 10^11^ viral genome per mouse and then maintained for 4 weeks, followed by a single intraperitoneal injection of DOX (15 mg/kg) for 8 days to generate DOX‐induced acute cardiotoxicity in mice. A, The IL‐1β and TNF‐α levels in myocardial tissues (n = 6). B and C, Representative western blot images and the corresponding statistical results (n = 6). (D) Representative images of CD45 staining in heart tissues (n = 6). E, Statistical results of M1‐type and M2‐type macrophages infiltration to the myocardium (n = 6). F, The mRNA level of M1‐type macrophage markers (*Il‐1β*, *Tnf‐α*, *iNos*, and *Cox‐2*), M2‐type macrophage markers (*Il‐10*, *Retnla*, *Arg‐1*, and *Cd163*) in murine hearts (n = 6). Values represent the mean ± SD. ^*^ *P *< .05 versus NS + NC, ^#^ *P *< .05 versus DOX+NC. In Figure 4E,F, ^*^ *P *< .05 versus the matched group

To explore the beneficial role of OSTN in vivo, we specifically overexpressed OSTN in murine hearts using the AAV9 system. As shown in Figure 5A, OSTN overexpression significantly decreased myocardial IL‐1β and TNF‐α levels in DOX‐treated mice. Consistently, DOX‐elicited the phosphorylation and nuclear translocation of P65 was markedly attenuated in OSTN‐overexpressed murine hearts (Figure 5B,C). DOX administration is reported to orchestrate a pro‐inflammatory tissue microenvironment in murine hearts and evokes leukocyte recruitment to the myocardium. Inspiring, CD45 staining suggested that the infiltration of leukocyte to murine hearts was blunted by OSTN overexpression (Figure 5D). Macrophage is identified as a key cell type in inflammation and its phenotypic polarization has been shown to have a profound impact on DOX‐elicited cardiac injury.^48^, ^49^ DOX is reported to promote M1 macrophage polarization and inhibit M2 macrophage recruitment in murine hearts, and we thus investigated the alteration of CD80/CD68‐positive M1 macrophages and CD206/CD68‐positive M2 macrophages in DOX‐treated murine hearts. Interesting, we observed that OSTN overexpression significantly decreased M1 macrophage polarization without affecting M2 macrophage infiltration after DOX injection (Figure 5E; Figure S1A). Consistently, the expressions of M1 markers, such as *Il‐1β*, *Tnf‐α*, and induced nitric oxide synthase (*iNos*), were decreased, whereas M2 markers, such as resistin‐like alpha (*Retnla*), arginase‐1 (*Arg‐1*), and *Cd163*, were not affected by OSTN overexpression in response to DOX (Figure 5F). A previous study showed that inhibition of cyclooxygenase‐2 (COX‐2), a key molecule for M1 polarization, remarkably attenuated the progression of DOX‐triggered cardiac dysfunction.^50^ Interestingly, no alteration of *Cox‐2* mRNA level was observed, but the anti‐inflammatory cytokine, *Il‐10* expression was increased by OSTN (Figure 5F). These results indicate that cardiac‐restricted overexpression of OSTN suppresses DOX‐induced inflammatory response in vivo.

### 3.6 OSTN blocks DOX‐induced oxidative stress and cardiomyocyte apoptosis in vivo

**FIGURE 6:**
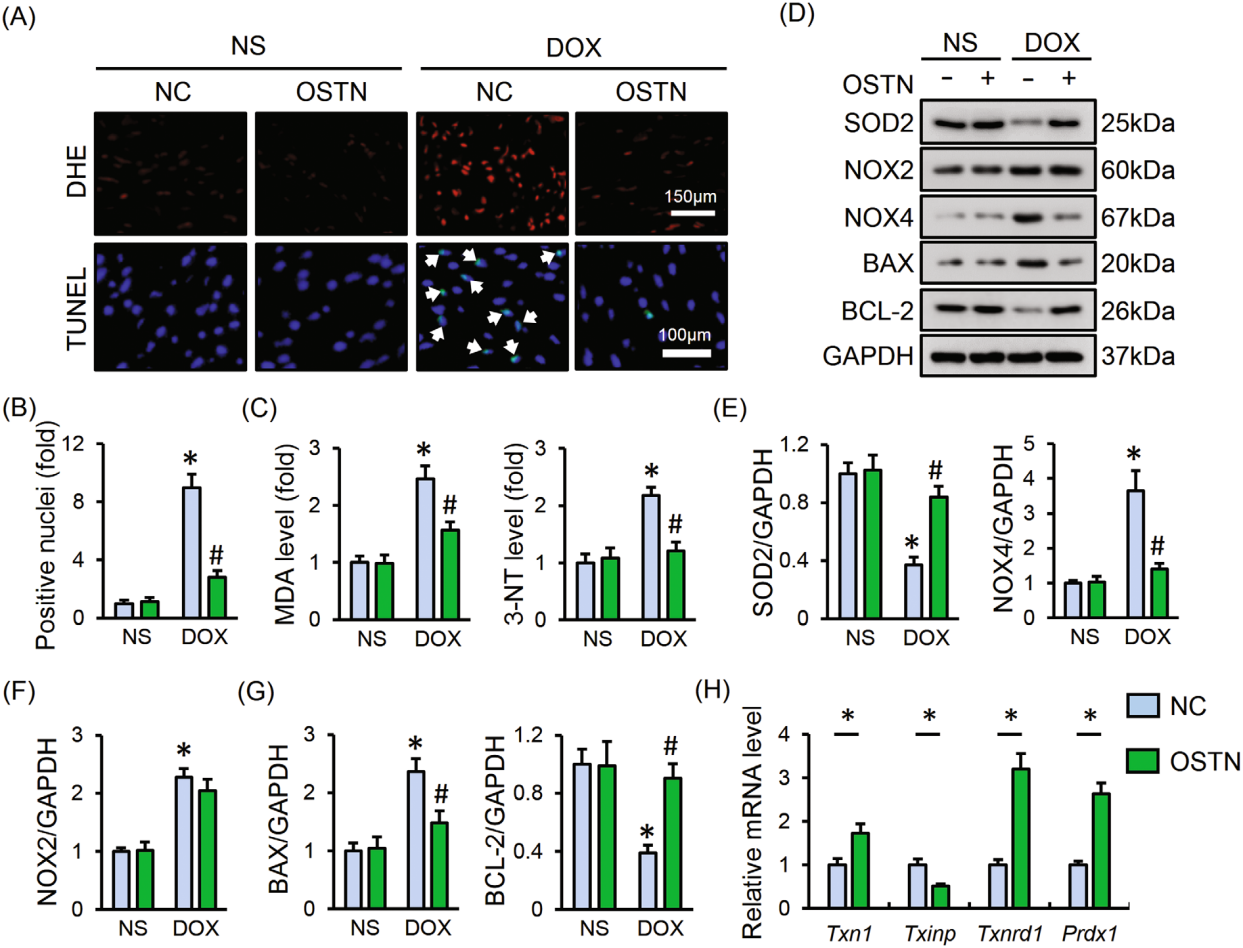
OSTN blocks DOX‐induced oxidative stress and cardiomyocyte apoptosis in vivo. A, Representative images of DHE and TUNEL staining in myocardium (n = 8). B, Quantitative results of TUNEL‐positive nuclei in heart tissues (n = 8). C, The level of MDA, 3‐NT in heart tissues (n = 6). D‐G, Representative western blot images and the corresponding statistical results (n = 6). H, The mRNA level of *Txn1*, *Txinp*, *Txnrd1*, and *Prdx1* in heart tissues (n = 6). Values represent the mean ± SD. ^*^ *P *< .05 versus NS + NC; ^#^ *P *< 0.05 versus DOX + NC. In Figure 6H, ^*^ *P *< .05 versus the matched group

As shown in Figure 6A,B, DOX treatment caused increases of ROS generation and cell apoptosis within the myocardium that were notably blocked by OSTN overexpression, as evidenced by the DHE and TUNEL staining. Besides, DOX‐triggered MDA and 3‐NT overproduction were also ameliorated in OSTN‐overexpressed murine hearts (Figure 6C). Protein analysis revealed that OSTN overexpression upregulated SOD2, BCL‐2 expression, while downregulated NOX4, BAX expression after DOX exposure, without affecting NOX2 protein level (Figure 6D‐G). Correspondingly, OSTN‐overexpressed mice exhibited increased SOD activity and decreased NOX, caspase3 activities (Figure S1B,C). In line with in vitro data, we also found that the disturbed thioredoxin system was markedly restored in murine hearts with OSTN overexpression, as confirmed by the increased *Txn1*, *Txnrd1*, and *Prdx1*, and decreased *Txinp* mRNA levels (Figure 6H). Mitochondria are identified as the major source of ROS within the heart and play indispensable roles in regulating DOX‐induced oxidative damage and apoptosis. Previous studies showed that OSTN acted to enhance the mitochondrial biogenesis, and we thus assessed mitochondrial alterations in DOX‐treated hearts with or without OSTN overexpression. As depicted in Figure S1D‐F, DOX treatment decreased mitochondrial content and function, which were notably improved by OSTN overexpression, as evidenced by the increased mtDNA level, mtProtein level, mtDNA integrity, and cardiac ATP level. The efficiency of AAV9‐mediated overexpression in heart samples was confirmed by western blot, while no alteration of circulating OSTN was observed (Figure S1G,H). These data provide further evidence that cardiac‐specific overexpression of OSTN blocks oxidative stress and cardiomyocyte apoptosis in DOX‐induced cardiotoxicity.

### 3.7 OSTN improves DOX‐induced cardiotoxicity in mice

**FIGURE 7:**
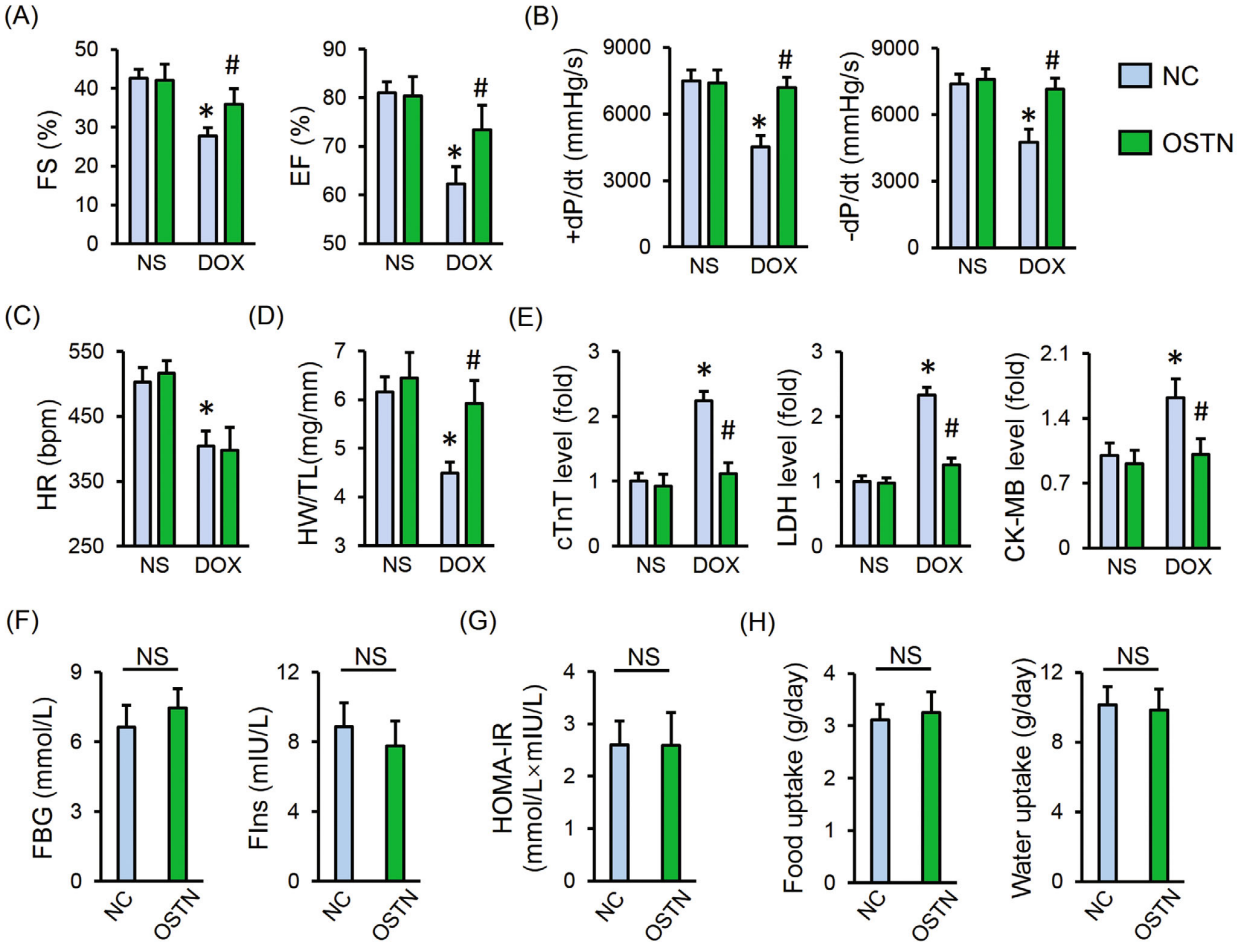
OSTN improves DOX‐induced acute cardiotoxicity in mice. A and B, Cardiac functional parameters of fractional shortening (FS), ejection fraction (EF), and the peak rates of isovolumic pressure development and pressure decay (±*dP*/*dt*) in left ventricles (n = 8). C, Heart rate (HR) (n = 8). D, Heart weight to tibial length ratio (HW/TL) (n = 8). E, Serum levels of cTnT, LDH, and CK‐MB in mice with or without OSTN overexpression after DOX injection (n = 8). F, Levels of fasting blood glucose (FBG) and fasting serum insulin (FIns) in control mice with or without OSTN overexpression (n = 8). G, The homeostasis model of assessment for insulin resistance (HOMA‐IR) index (n = 8). H, Food uptake and water uptake in control mice with or without OSTN overexpression (n = 10). Values represent the mean ± SD. ^*^ *P *< .05 versus NS+NC; ^#^ *P *< .05 versus DOX + NC; NS, no significance

Given the fact that OSTN shows remarkably beneficial effect on DOX‐elicited inflammation, oxidative stress, and cardiomyocyte apoptosis in vivo and in vitro, we hence investigated whether OSTN overexpression could mitigate cardiac injury and malfunction upon DOX insult. As shown in Figure 7A,B, mice injected with AAV9‐OSTN exhibited an improvement in cardiac function during acute DOX administration, as confirmed by the increased fractional shortening (FS), ejection fraction (EF), and the peak rates of isovolumic pressure development and pressure decay (±*dP*/*dt*) in left ventricles. Yet, no alteration of heart rate (HR) was observed by OSTN overexpression in mice with or without DOX injection (Figure 7C). Of note, OSTN overexpression alone did not affect cardiac function under physiological conditions (Figure 7A‐C). Besides, we also found that DOX‐triggered heart weight (HW) loss was also improved, as confirmed by the increased HW/tibial length (TL) (Figure 7D). We further measured the level of serum biomarkers related to cardiac injury, and found that serum levels of cTnT, LDH, and CK‐MB were all decreased by OSTN overexpression (Figure 7E). Nishizawa et al previously identified that recombinant OSTN treatment might cause insulin resistance via inhibiting insulin‐stimulated glycogen synthesis and glucose uptake in skeletal myocytes.^51^ Inspiringly, there was no difference on FBG, FIns, and HOMA‐IR between mice with or without a cardiac‐specific overexpression of OSTN (Figure 7F,G). Food and water uptake were also not affected (Figure 7H).

To enhance the clinical impact of our study, we also assessed the cardioprotective capacity of OSTN during chronic DOX treatment. As depicted in Figure S2A‐C, cardiac dysfunction in mice with repeated DOX injections was remarkably alleviated by OSTN overexpression, as confirmed by the improved FS, EF, and ±*dP*/*dt*. Besides, we observed that chronic DOX injections caused a significant decrease of HW/TL in the control mice, but to a less extent in that with OSTN protection (Figure S2D). More importantly, OSTN overexpression reduced the mortality rate in the chronic model of DOX‐induced cardiotoxicity (Figure S2E). These results identify OSTN as a negative regulator of DOX‐induced cardiotoxicity.

### 3.8 OSTN prevents DOX‐induced inflammation, oxidative stress, and apoptosis via activating PKG

**FIGURE 8:**
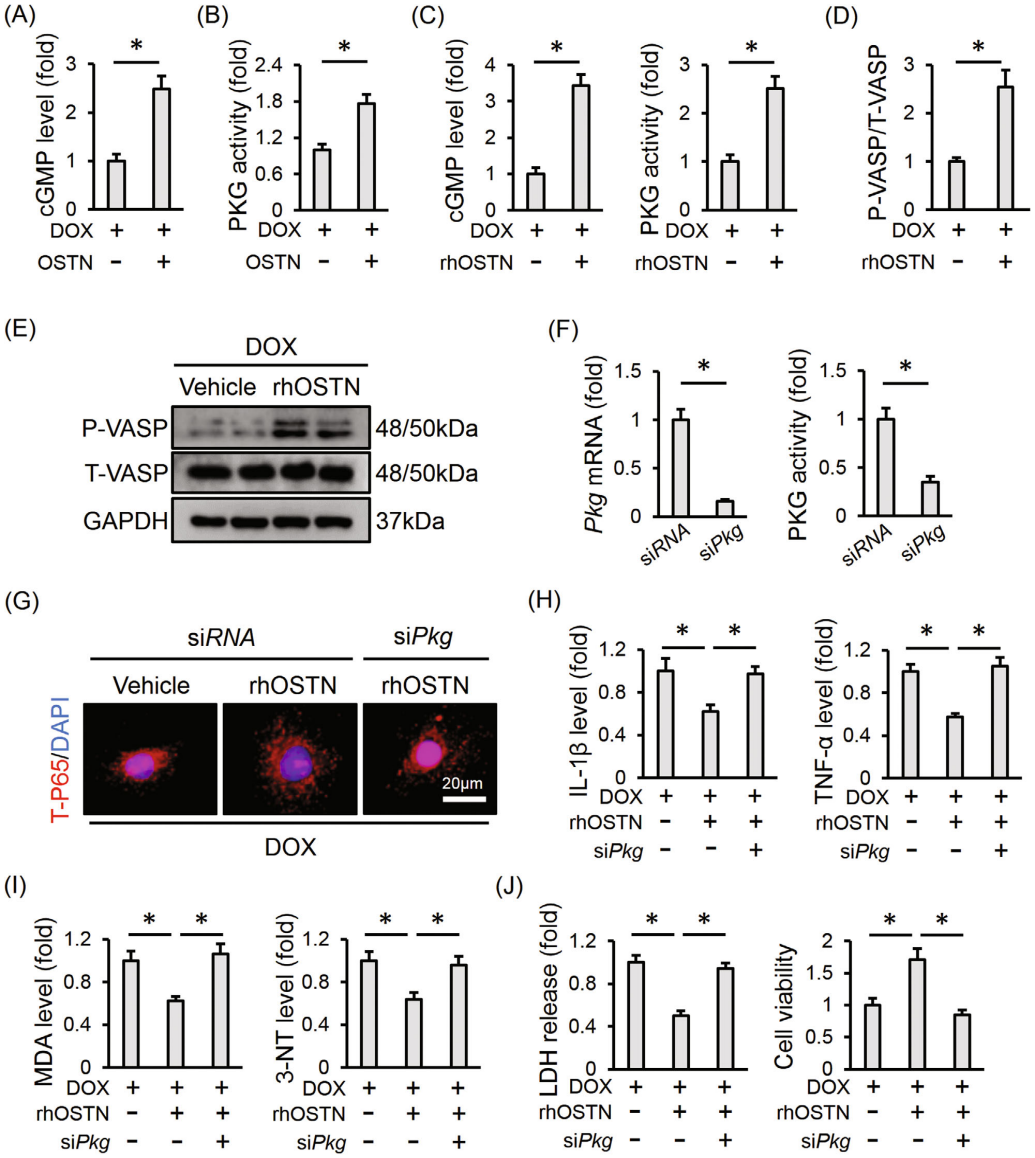
OSTN prevents DOX‐induced inflammation, oxidative stress, and apoptosis via activating PKG. A, Myocardial cGMP level with or without OSTN overexpression after DOX injection (n = 6). B, Relative PKG activity in the myocardium (n = 6). C, Relative cGMP level and PKG activity in DOX‐treated H9C2 cells with or without rhOSTN incubation for 24 h (n = 6). D and E, Representative western blot images and the corresponding statistical results in H9C2 cells with or without rhOSTN incubation (n = 6). F, H9C2 cells were incubated with si*Pkg* (50 nmol/L) or si*RNA* (50 nmol/L) for 4 h and then maintained in normal medium for additional 24 h. The efficiency of si*Pkg* in H9C2 cells was detected by western blot (n = 6). G, H9C2 cells were pre‐infected with si*Pkg* or si*RNA*, and then received DOX insult (1µmol/L) with or without rhOSTN protection (5 µg/mL). Immunofluorescence staining of T‐P65 in H9C2 cells (n = 6). H, The releases of IL‐1β and TNF‐α from H9C2 cells to the medium (n = 6). I, The levels of MDA and 3‐NT in H9C2 cells (n = 6). J, LDH release from H9C2 cells and the quantitative data of cell viability (n = 6). Values represent the mean ± SD. ^*^ *P *< .05 versus the matched group

OSTN was previously defined as an endogenous regulator of natriuretic peptides system and acted to locally modulate cGMP homeostasis in the bone and skeletal muscle.^20^, ^22^ Interestingly, we observed that OSTN overexpression significantly increased myocardial cGMP level in response to DOX injection (Figure 8A). Besides, PKG, the primary and crucial downstream target of cGMP, was also activated by OSTN overexpression in DOX‐treated murine hearts (Figure 8B). Moreover, H9C2 cells treated with rhOSTN exhibited increased cGMP levels and PKG activity, which was further corroborated by the increased phosphorylation of VASP, a downstream target of PKG (Figure 8C‐E). To validate the role of PKG in OSTN‐mediated protective effect in vitro, H9C2 cells were pretreated with si*Pkg* to knock down the expression of PKG, and the efficiency was confirmed by the PCR data together with enzymatic activity (Figure 8F). As depicted in Figure 8G, rhOSTN incubation notably inhibited DOX‐triggered P65 nuclear translocation, which was abolished in *Pkg*‐deficient cells. Meanwhile, the inhibitory effect of rhOSTN on the releases of IL‐1β and TNF‐α was also blocked with PKG knockdown (Figure 8H). In addition, PKG silence abrogated rhOSTN‐mediated beneficial effect on productions of MDA and 3‐NT (Figure 8I). The aberrant enzymatic activities of SOD and NOX were restored by rhOSTN in si*RNA*‐treated cells, but not in that infected with si*Pkg* (Figure S3A). As expected, the reduced LDH release, caspase3 activity, and enhanced cell viability seen in rhOSTN‐treated H9C2 cells were almost completely abolished by PKG knockdown (Figure 8J; Figure S3B). Further detection with DCFH‐DA and TUNEL staining revealed that the protective effect of rhOSTN on DOX‐elicited oxidative stress and apoptosis was mediated by PKG activation in vitro (Figure S3C). These results define PKG as the potential molecular target for OSTN‐mediated anti‐inflammatory, anti‐oxidant, and anti‐apoptotic capacities upon DOX insult.

### 3.9 PKG inhibition abolishes the beneficial effect of OSTN in vivo

**FIGURE 9:**
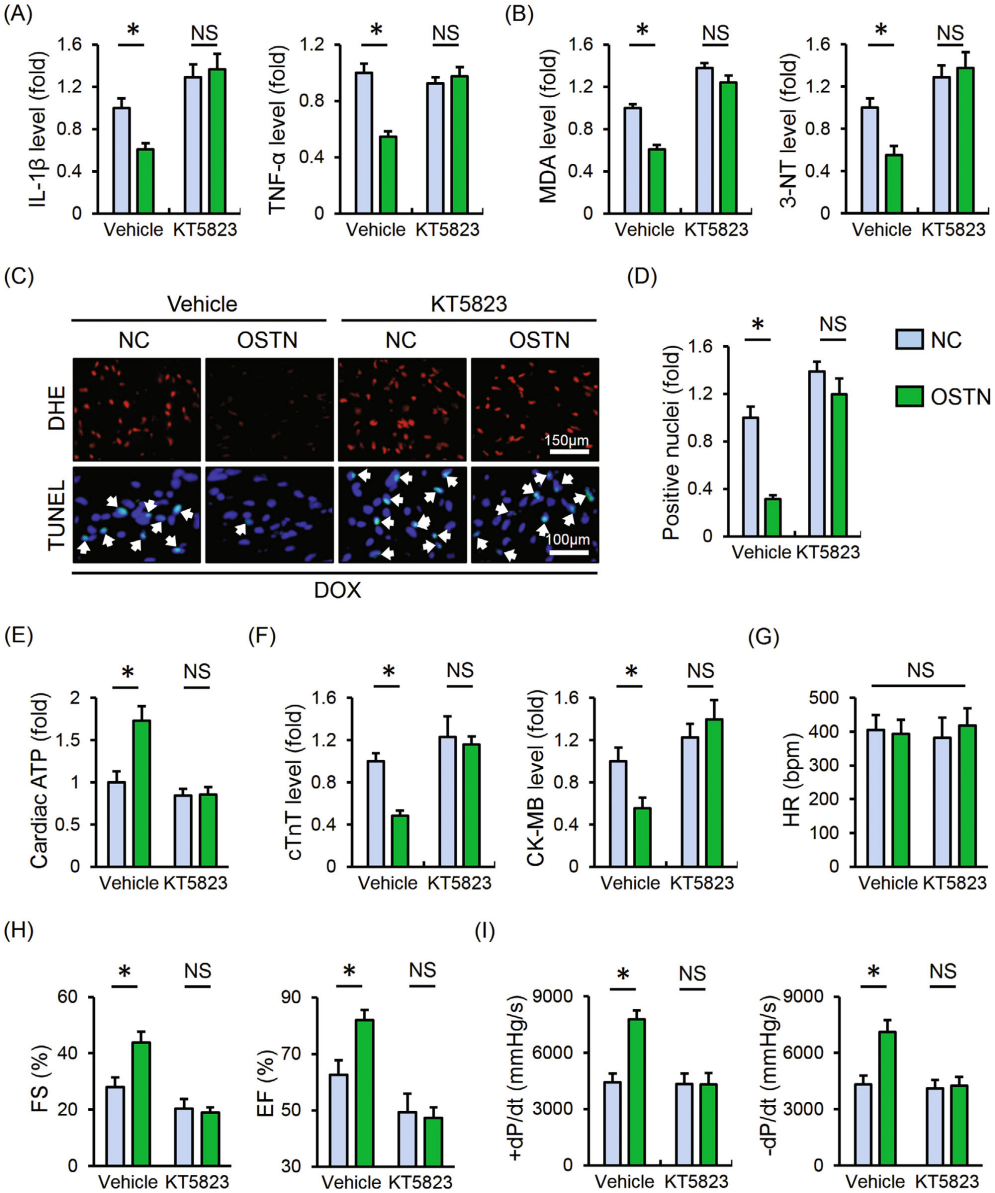
PKG inhibition abolishes the beneficial effect of OSTN in vivo. After AAV9 treatment, mice were intraperitoneally injected with KT5823 (1 mg/kg/day) or an equal volume of vehicle for consecutive 3 days prior to DOX treatment. A, The IL‐1β and TNF‐α levels in myocardial tissues (n = 6). B, The level of MDA and 3‐NT in heart samples (n = 6). C, Representative images of DHE and TUNEL staining in the myocardium (n = 8). D, Quantitative results of TUNEL‐positive nuclei in heart tissues (n = 8). E, Cardiac adenosine triphosphate (ATP) level in mice (n = 6). F, Serum levels of cTnT and CK‐MB in mice (n = 6). G, HR in mice among groups (n = 8). H and I, The statistical data of FS, EF, and ±*dP*/*dt* (n = 8). Values represent the mean ± SD. ^*^ *P *< .05 versus the matched group; NS, no significance

Mice were then injected with KT5823 to further assess the involvement of PKG. As shown in Figure 9A, OSTN overexpression significantly suppressed myocardial IL‐1β and TNF‐α levels in DOX‐injected mice, yet failed to do so in that treated with PKG inhibitor. The anti‐oxidant and anti‐apoptotic effects of OSTN were absolutely repressed in the presence of KT5823, as determined by the unaltered MDA, 3‐NT levels, and DHE, TUNEL staining (Figure 9B‐D). OSTN overexpression‐mediated the increase of cardiac ATP level was also decreased after PKG suppression (Figure 9E). In addition, OSTN markedly reduced serum level of cTnT and CK‐MB in DOX‐injured mice, which was abrogated in mice with PKG inhibition (Figure 9F). More importantly, OSTN overexpression failed to improve DOX‐induced cardiac dysfunction in mice treated with PKG inhibitor and no alteration of HR was observed among groups (Figure 9G‐I). All these findings prove that PKG inhibition abolishes the beneficial effect of OSTN in vivo.

## 4 DISCUSSION

In this study, we demonstrate that OSTN supplementation protects against DOX‐induced cardiotoxicity, which is presumably associated with its anti‐inflammatory, anti‐oxidant, and anti‐apoptotic actions. Mechanistically, we identify that OSTN activates PKG, and PKG inhibition blocks the beneficial effect of OSTN in vivo and in vitro. Collectively, our data define OSTN as a potential therapeutic target for DOX‐induced cardiotoxicity.

DOX shows a high affinity to heart tissues and can be abundantly accumulated in the cardiomyocytes, which eventually causes cell loss and life‐threatening cardiac dysfunction even at low cumulative dosage.^9^ To date, there is no effective interventions against DOX‐induced cardiotoxicity except for dexrazoxane. Aerobic exercise is regarded as an availably nonpharmacological therapy for DOX‐induced cardiotoxicity with no disturbance on its chemotherapeutic efficacy.^52^, ^53^, ^54^ Jensen et al showed that exercise preconditioning also reduced myocardial DOX accumulation, thereby preventing cardiac dysfunction in rats.^55^ However, chemotherapy and tumor itself can decrease skeletal muscle mass and induce cachexia as well as cardiopulmonary dysfunction, which remarkably compromise physical endurance in cancer patients.^56^ In addition to being a motor organ, muscle cells are thought to have the capacity to produce and release a variety of cytokines or peptides, named myokines that play crucial roles in mediating the health benefits of exercise in a hormone‐like fashion.^57^, ^58^ Therefore, targeting these myokines might help to counteract DOX‐induced cardiotoxic effects, especially for cancer patients who cannot bear the general exercise.^59^ A previous study determined that the myokine, fibroblast growth factor 21 obviously attenuated DOX‐induced cardiac dysfunction and pathologic alterations.^60^ Besides, our recent study revealed that the other myokine, FNDC5/Irisin supplementation prevented oxidative stress, cardiomyocyte apoptosis, and cardiac dysfunction in DOX‐treated mice.^5^ Results from Suzuki et al demonstrated that intracoronary delivery of skeletal myoblasts significantly improved cardiac function and survival rate in mice upon DOX injury. These beneficial effects might be associated with the secretion of a certain growth factors, and they proposed that gene therapy with myoblasts‐expressing proteins was capable of enhancing cardiomyocyte contraction.^61^ Accordingly, we herein observed that cardiac gene therapy with a myokine, OSTN prevented DOX‐induced inflammation, oxidative stress, apoptosis, and cardiac dysfunction. Moreover, Re and colleagues lately found that OSTN retarded muscle atrophy in cancer cachexia mice and adapted the skeletal muscle to aerobic exercise.^62^ Collectively, OSTN could be a promising cardioprotective factor for cancer patients exposed to DOX treatment or suffered to cachexia.

Despite the therapeutic potential in DOX‐induced cardiotoxicity, systematic exposure to OSTN may cause multiple peripheral effects on bone or skeletal muscle.^16^ Nishizawa et al showed that an increased OSTN level might be related to insulin resistance via inhibiting insulin‐stimulated glycogen and synthesis glucose uptake in skeletal myocytes.^51^ In the current study, we observed a certain degree of OSTN expression within the heart under physiological conditions that was decreased upon DOX treatment, addressing the possibility to treat DOX‐induced cardiotoxicity via replenishing myocardial OSTN specially. Gene therapy provides targeting, durable and radical benefits in contrast to protein‐based drugs or cellular transplantation with unstable effects and complex manipulation, which has emerged as a vital component of the therapeutic armamentarium for the management of multiple cardiovascular diseases.^63^, ^64^ There are several completed or ongoing clinical trials of cardiac gene therapy, such as CUPID1/2, STOP‐HF, RETRO‐HF, and so on.^64^, ^65^ A great deal of both viral vectors such as adenovirus, lentivirus, and retrovirus, in addition to non‐viral delivery methods such as lipid vesicles and naked plasmid DNA, is currently being tested. For most gene delivery systems, recombinant parvovirus AAVs are identified as an attractive choice in the gene therapy community due to the small size, cell‐cross capacity, nonpathogenic characteristic, and sustained gene‐transfer efficiency.^65^, ^66^ Refinements in vector technology and the intensive understanding about viral nature have allowed for safe and efficient gene transfer to the myocardium using AAVs. AAV9 is a cardiotropic vector that has the best viral genome distribution and highest protein levels after systematic injection.^67^, ^68^ These features propose an intravenous delivery of AAV9 instead of intramyocardial or coronary injections that are related with additional invasive injuries. The high affinity and gene‐transfer efficiency of AAV9 help to increase its overall safety by reducing the concentrations of viral particles and capsid proteins. Besides, the decreased amounts of viral particles further avoid the likelihood of host genome integration.^66^ Inspiringly, Pacak et al found no side effects or severe adverse events in mice or primates following systemic gene delivery by AAV9.^66^ Considering these effects, we used AAV9 to overexpress OSTN in the myocardium by tail vein injection. In addition to the heart, skeletal muscle and other tissues are also targets of AAV9 after systemic delivery. To avoid the possible adverse effects, we cloned OSTN gene under the control of a cardiac‐specific promoter and found that circulating OSTN level was unaffected by such a vector. Accordingly, no alteration on insulin sensitivity and uptake of food/water was observed during the study. Our data provide a novel therapeutic strategy using a clinically feasible delivery route for treating DOX‐induced cardiotoxicity.

Relatively little is known about how OSTN exerts its beneficial effects. Herein, we proved that the cardioprotective effect of OSTN against DOX‐related cardiotoxicity was mediated by PKG, while PKG inhibition abolished the protective effect of OSTN on inflammation, oxidative stress, and apoptosis in DOX‐treated mice or cells. PKG activation is reported to increase myocardial hydrogen sulfide production that is an endogenous cardioprotective molecule against DOX‐induced cardiotoxicity.^29^, ^69^, ^70^ Besides, prophylactic treatment with an indirect PKG activator, sildenafil not only attenuated DOX‐induced cardiac dysfunction but also increased its chemotherapeutic efficacy in mice.^25^, ^26^, ^27^ Consistent with our current study, previous studies proved that PKG activation inhibited P65 nuclear translocation and inflammatory cells infiltration, thereby decreasing the production of inflammatory cytokines.^71^, ^72^ PKG is also implicated in the regulation of redox homeostasis via upregulating various anti‐oxidant genes, and then alleviates oxidative stress‐induced cell injury in vivo and in vitro.^24^, ^73^, ^74^ In addition, we found that OSTN‐mediated PKG activation restored myocardial thioredoxin system disturbed by DOX, which is in line with a recent study,^75^ All these data prove that PKG might be a therapeutic target against DOX‐induced cardiac dysfunction. PKG can be activated by at least by two independent pathways, either by the formation of a cysteine‐42 (Cys‐42)‐dependent intermolecular disulfide or through a cGMP‐mediated allosteric modulation.^25^, ^76^ Upon oxidative stress, PKG forms an inter‐disulfide between Cys‐42 that functions as a compensatory protection mechanism to increase the kinase activity independently of cGMP.^76^ However, Prysyazhna et al proved that DOX‐elicited PKG disulfide dimerization probably promoted cardiomyocyte apoptosis and cardiac dysfunction.^25^ Numerous studies determined that oxidant‐induced disulfide formation in PKG was precluded by cGMP‐dependent modulation, accompanied by an increased activation of RhoA and the downstream pro‐survival pathways.^25^, ^77^ Based on these data, we reasonably speculate that the cardioprotective effect of OSTN during DOX treatment is mediated, at least in part, via elevating the cGMP level and PKG activity.

## 5 CONCLUSIONS

Taken together, our data imply that OSTN treatment suppresses DOX‐induced inflammation, oxidative stress, apoptosis, and cardiac dysfunction via activating PKG. OSTN could be identified as a potent therapeutic agent for DOX‐induced cardiotoxicity.

## CONFLICT OF INTEREST

The authors declare no conflict of interest.

## ETHICS APPROVAL AND CONSENT TO PARTICIPATE

All animal experiments were performed in strict accordance with the *Guidelines for the Care and Use of Laboratory Animals* (NIH Publication, revised 2011), which were also approved by the Animal Care and Use Committee of Renmin Hospital of Wuhan University.

## AUTHOR CONTRIBUTIONS

Can Hu, Xin Zhang, and Qi‐Zhu Tang contributed to the conception and design of the experiment. Can Hu, Xin Zhang, Ning Zhang, and Wen‐Ying Wei performed the study and acquired data. Can Hu, Xin Zhang, and Ling‐Li Li made data analysis and contributed to the data interpretation. Can Hu and Xin Zhang drafted and revised the manuscript. Zhen‐Guo Ma and Qi‐Zhu Tang were responsible for the financial support, study supervision, and final approval of manuscript.

## Supporting information

Supporting information

## Data Availability

All data that support the findings in this study are available from the corresponding author upon reasonable request.
