# Supplementary material for: Osteocrin attenuates inflammation, oxidative stress, apoptosis, and cardiac dysfunction in doxorubicin‐induced cardiotoxicity
Source: Clin Transl Med. 2020 Jul 3;10(3):e124. doi: 10.1002/ctm2.124 (PMC7418805; doi:10.1002/ctm2.124)
Supplement: Supplementary file 1 — Supporting information [file CTM2-10-e124-s001.docx]

**Supporting information**

**Osteocrin attenuates inflammation, oxidative stress, apoptosis and cardiac dysfunction in doxorubicin-induced cardiotoxicity**

Can Hu^1, 2^ ^*^, Xin Zhang^1, 2^ ^*^, Ning Zhang^1, 2^, Wen-Ying Wei^1, 2^, Ling-Li Li^1, 2^

Zhen-Guo Ma^1, 2^ & Qi-Zhu Tang^1, 2^

^1^ Department of Cardiology, Renmin Hospital of Wuhan University, Wuhan 430060, China

^2^ Hubei Key Laboratory of Metabolic and Chronic Diseases, Wuhan 430060, China

^*^ These authors contributed equally to this work.

Corresponding author:

**Qi-Zhu Tang** and **Zhen-Guo Ma**,

Department of Cardiology,

Renmin Hospital of Wuhan University,

Hubei Key Laboratory of Metabolic and Chronic Diseases,

Wuhan University at Jiefang Road 238, Wuhan 430060, PR China

Tel.: +86 027-88073385; Fax: +86 027-88042292.

E-mail: [qztang@whu.edu.cn](mailto:qztang@whu.edu.cn) (Qi-Zhu Tang) and [zhengma@whu.edu.cn](mailto:zhengma@whu.edu.cn) (Zhen-Guo Ma).

Running title: Osteocrin attenuates doxorubicin-induced cardiotoxicity.

The authors declared no conflict of interest.


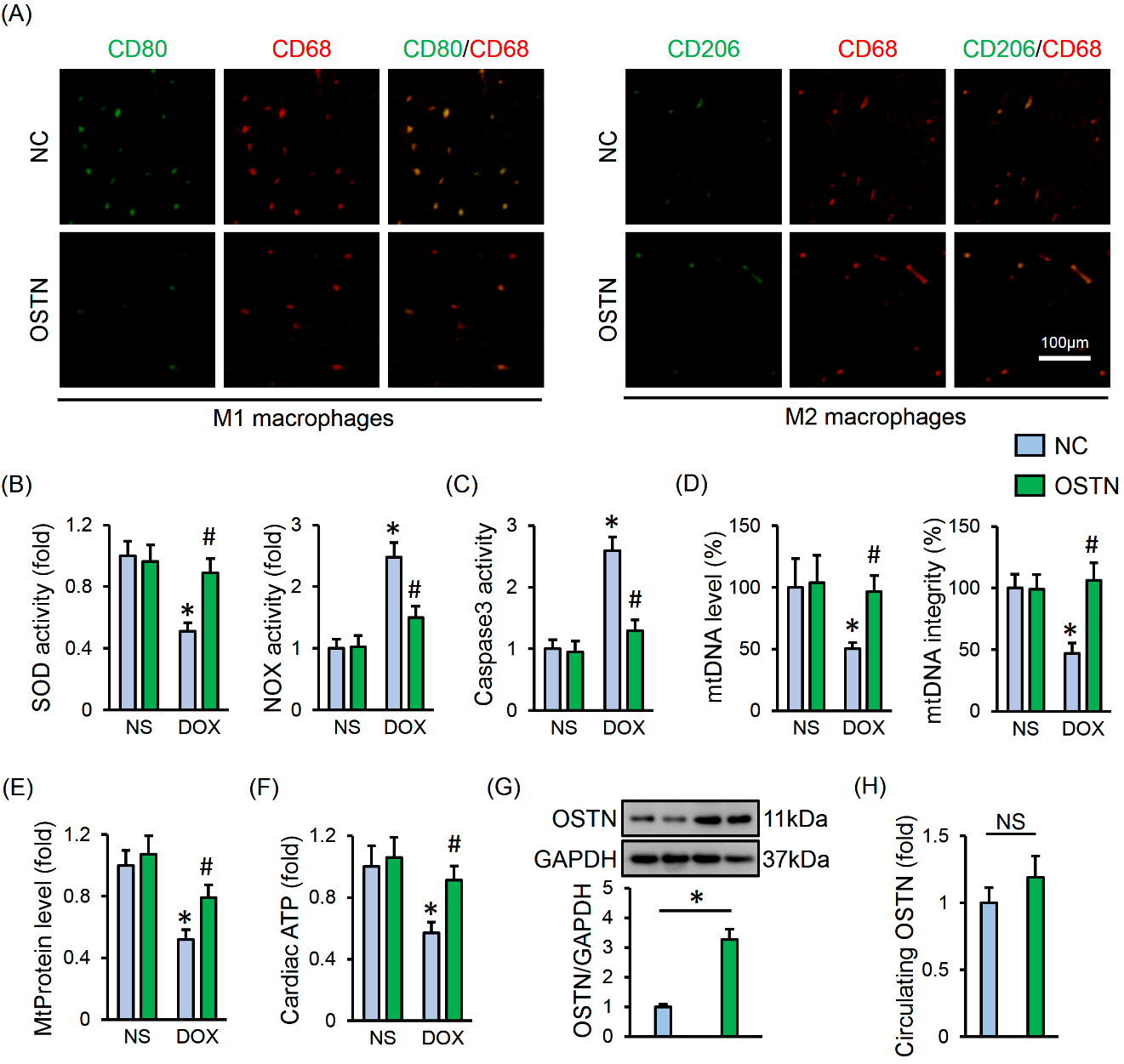


**Figure S1. OSTN prevents DOX-induced inflammation, oxidative stress and apoptosis in mice.** Mice were exposed to a single intravenous injection of AAV9-OSTN (OSTN) or AAV9-NC (NC) at a dosage of 1×10^11^ viral genome per mouse and then maintained for 4 weeks, followed by a single intraperitoneal injection of DOX (15mg/kg) for 8 days to generate DOX-induced acute cardiotoxicity in mice. **(A)** Representative immunofluorescence images of CD80/CD68-positive M1 macrophages and CD206/CD68-positive M2 macrophages in DOX-treated murine hearts (n=6). **(B)** Relative SOD and NOX activity in the myocardium (n=6). **(C)** Caspase3 activity in murine hearts from indicating groups (n=6). **(D)** Quantification of mtDNA content and integrity (n=6). **(E)** Quantification of mtProtein level (n=6). **(F)** Statistical data of cardiac ATP level (n=6). **(G)** Four weeks post-AAV9 injection, the efficiency of OSTN overexpression was determined by western blot (n=6). **(H)** Circulating OSTN level in mice with or without AAV9-OSTN injection for 4 weeks (n=8). Values represent the mean±SD. **P*<0.05 versus NS+NC, #*P*<0.05 versus DOX+NC.


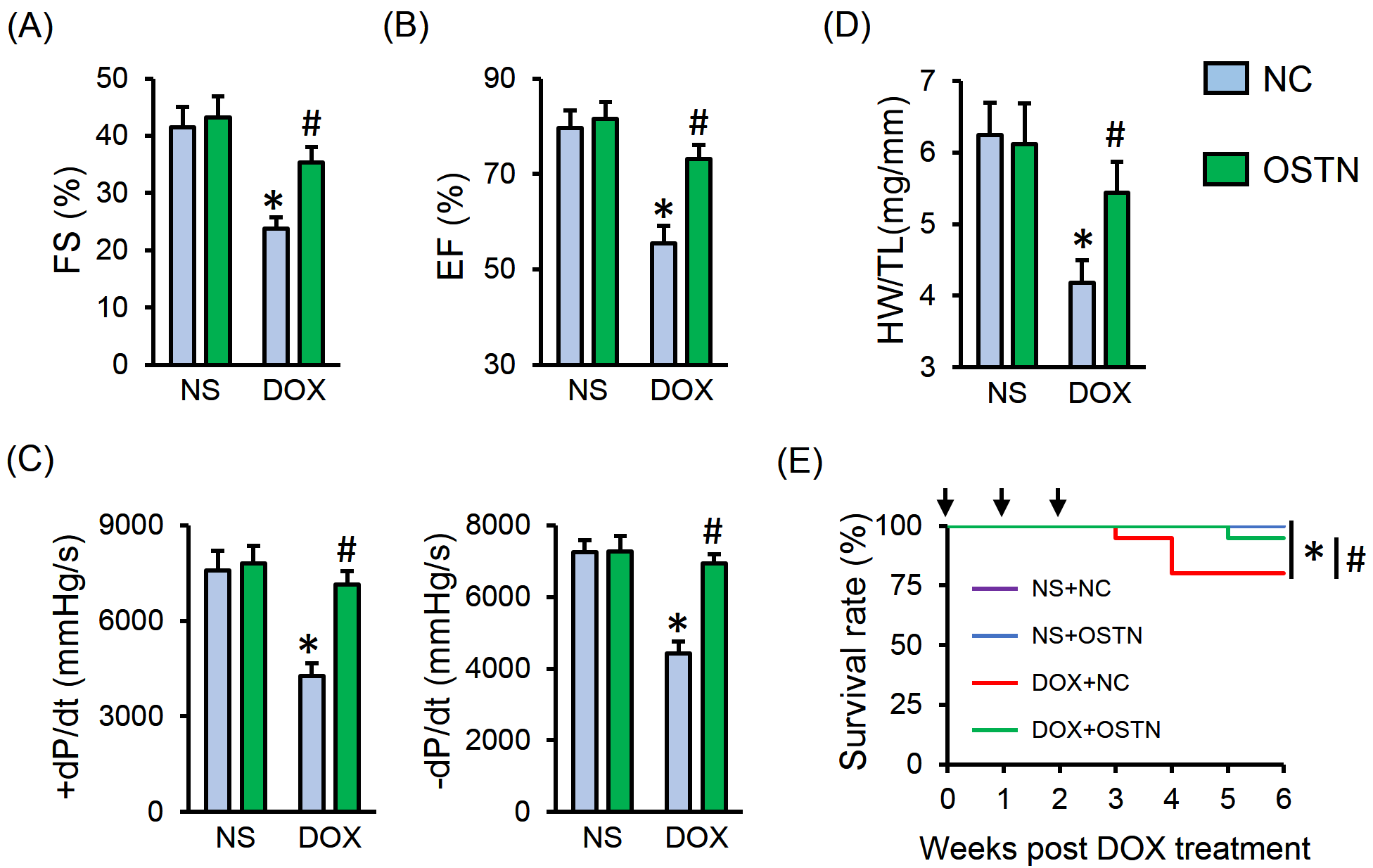


**Figure S2. OSTN improves DOX-induced chronic cardiotoxicity in mice.** Mice were exposed to a single intravenous injection of AAV9-OSTN (OSTN) or AAV9-NC (NC) at a dosage of 1×10^11^ viral genome per mouse and then maintained for 4 weeks. Next, mice received repeated injections of DOX (5mg/kg, once a week for consecutive 3 weeks) at a cumulative dose of 15mg/kg to generate a chronic model of DOX-induced cardiotoxicity and kept for additional 4 weeks after the last DOX injection. **(A-C)** Cardiac functional parameters of fractional shortening (FS), ejection fraction (EF) and the peak rates of isovolumic pressure development and pressure decay (±dP/dt) in left ventricles (n=8). **(D)** Heart weight to tibial length ratio (HW/TL) (n=8). **(E)** Survival rate in a chronic model of DOX-induced cardiotoxicity (n=20). Values represent the mean±SD. **P*<0.05 versus NS+NC, #*P*<0.05 versus DOX+NC.


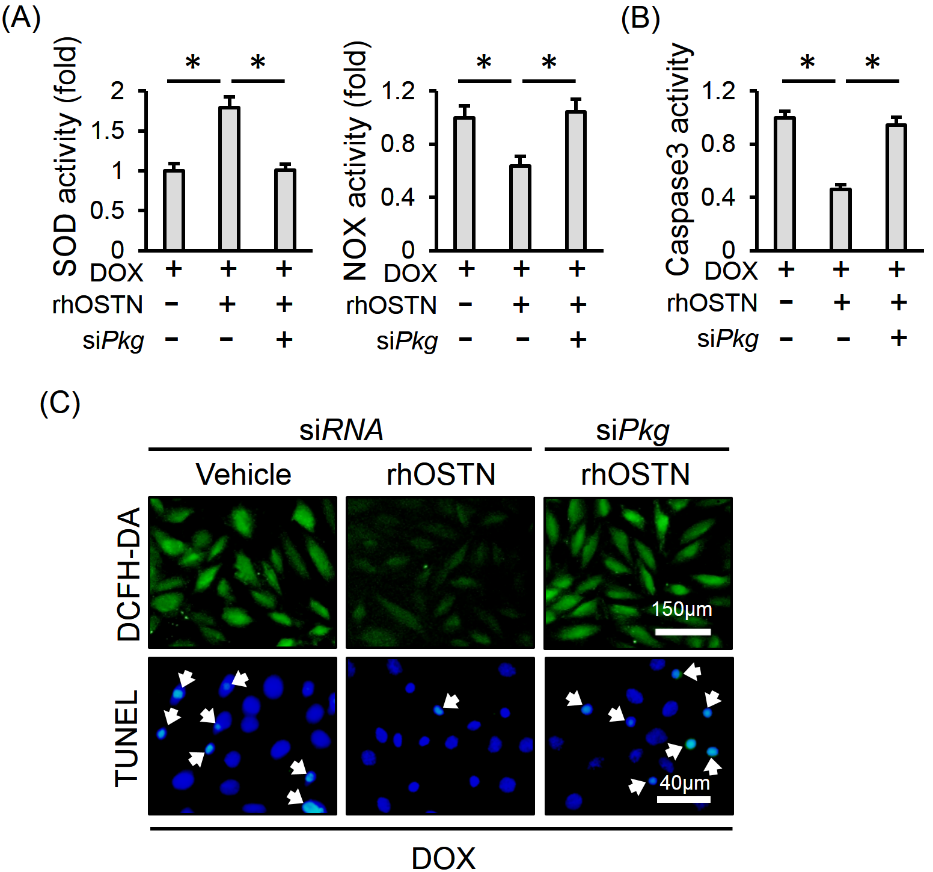


**Figure S3. *Pkg* deficiency abolished the beneficial effect of rhOSTN in vitro.** H9C2 cells were incubated with si*Pkg* (50nmol/L) or si*RNA* (50nmol/L) for 4 hours and then maintained in normal medium for 24 hours. Next, the cells received DOX insult (1μmol/L) with or without rhOSTN protection (5μg/ml) additional 24 hours. **(A)** SOD and NOX activity in H9C2 cells (n=6). **(B)** Caspase3 activity in H9C2 cells (n=6). **(C)** Representative images of DCFH-DA and TUNEL stating (n=6). Values represent the mean±SD. **P*<0.05 versus the matched group.
